# Long‐Acting Therapeutics in Pediatric Health: Bridging Innovation and Access

**DOI:** 10.1002/cpt.70458

**Published:** 2026-08-28

**Authors:** Prajith Venkatasubramanian, Moherndran Archary, Daniel B. Hawcutt, Rachel Daley, Edmund Capparelli, Adeniyi Olagunju

**Affiliations:** ^1^ Centre of Excellence for Long‐acting Therapeutics University of Liverpool Liverpool UK; ^2^ Department of Biochemistry, Cell and Systems Biology, Institute of Systems, Molecular and Integrative Biology University of Liverpool Liverpool UK; ^3^ Department of Pediatrics and Child Health, African Health Research Institute, University of KwaZulu‐Natal Victoria Mxenge Hospital Durban South Africa; ^4^ Alder Hey Research Division and NIHR Alder Hey Clinical Research Facility Alder Hey Children's NHS Foundation Trust Liverpool UK; ^5^ Department of Women's and Children's Health University of Liverpool Liverpool UK; ^6^ Skaggs School of Pharmacy and Pharmaceutical Sciences University of California San Diego La Jolla California USA; ^7^ Rady Children's Hospital San Diego San Diego California USA

## Abstract

Children have distinct therapeutic needs arising from age‐dependent physiology, disease epidemiology, formulation requirements, dosing considerations, and safety vulnerabilities. Despite substantial reductions in childhood mortality over the past three decades, nearly 5 million children under five died in 2023, with infectious diseases continuing to contribute substantially to preventable morbidity and mortality. Long‐acting therapeutics (LATs) may help address selected pediatric health priorities by sustaining therapeutic or prophylactic exposure, reducing dosing frequency, and improving adherence and implementation feasibility. This review synthesizes insights from the pediatric health session of the Community of Practice for Long‐acting Therapeutics for Maternal and Paediatric Health workshop. The session considered priority pediatric conditions in which existing or emerging LAT approaches could have clinical or public health impact, including lower respiratory tract infections, malaria, tuberculosis, HIV, and syphilis, as well as selected noninfectious indications such as childhood cancer and growth hormone deficiency. We then examine clinical pharmacology and safety considerations central to pediatric LAT development, including developmental pharmacokinetics, absorption‐limited disposition and flip‐flop kinetics, pharmacokinetic tailing, dose selection, model‐informed development, and injection‐site tolerability. The review also discusses long‐acting technology platforms with potential pediatric applications, route‐of‐administration considerations, excipient safety, formulation design, acceptability, and implementation challenges. Finally, we propose recommendations to support pediatric LAT development, including early integration of developmental pharmacology, age‐ and weight‐informed dosing, standardized acceptability assessment, product‐attribute frameworks, regulatory engagement, and equitable access planning. Realizing the potential of LATs for children will require coordinated, child‐centered, and equity‐driven approaches that address evidence generation, formulation suitability, implementation context, and affordability from the outset.

Despite substantial reductions in global childhood mortality, preventable infectious diseases remain major contributors to pediatric morbidity and mortality, particularly in low‐ and middle‐income countries (LMIC). In 2023, 4.8 million children died before age five, including 2.3 million newborns. These deaths are concentrated in fragile settings where access to health care, nutrition, and social protection remains limited. Infectious diseases such as lower respiratory tract infections (LRTIs), malaria, tuberculosis, human immunodeficiency virus (HIV), and syphilis remain important contributors.^1^

Long‐acting therapeutics (LATs) are increasingly being developed across clinical indications, but infants and children remain at risk of delayed or inequitable access to innovations first evaluated in adults. The Community of Practice for Long‐acting Therapeutics for Maternal and Paediatric Health, hosted by the University of Liverpool Centre of Excellence for Long‐Acting Therapeutics (CELT) within CELT Global Health, was established to support evidence generation, stakeholder engagement, and policy‐relevant outputs for LAT development in maternal and pediatric populations, including in LMICs. The Community of Practice brings together more than 80 members from globally representative cross‐sector stakeholder groups.

This review synthesizes insights from the pediatric health session of the July 1, 2025 workshop of the Community of Practice convened by CELT in Liverpool. The session comprised three linked presentations on pediatric health priorities, pharmacokinetic and safety considerations for LAT use in children, and long‐acting technologies with potential pediatric applications, followed by a moderated panel discussion and hybrid audience Q&A (**Figure** **1**). A published white paper has summarized cross‐cutting gaps in clinical research, access, and regulation across maternal and pediatric LAT development,^2^ while a related maternal health review has addressed priorities, efficacy and safety considerations, and emerging technologies for LAT use during pregnancy and lactation.^3^ Building on these, this article provides a pediatric‐focused synthesis of priority disease areas and the clinical pharmacology, formulation, safety, acceptability, and implementation considerations needed to advance equitable LAT development for children.

**Figure 1:**
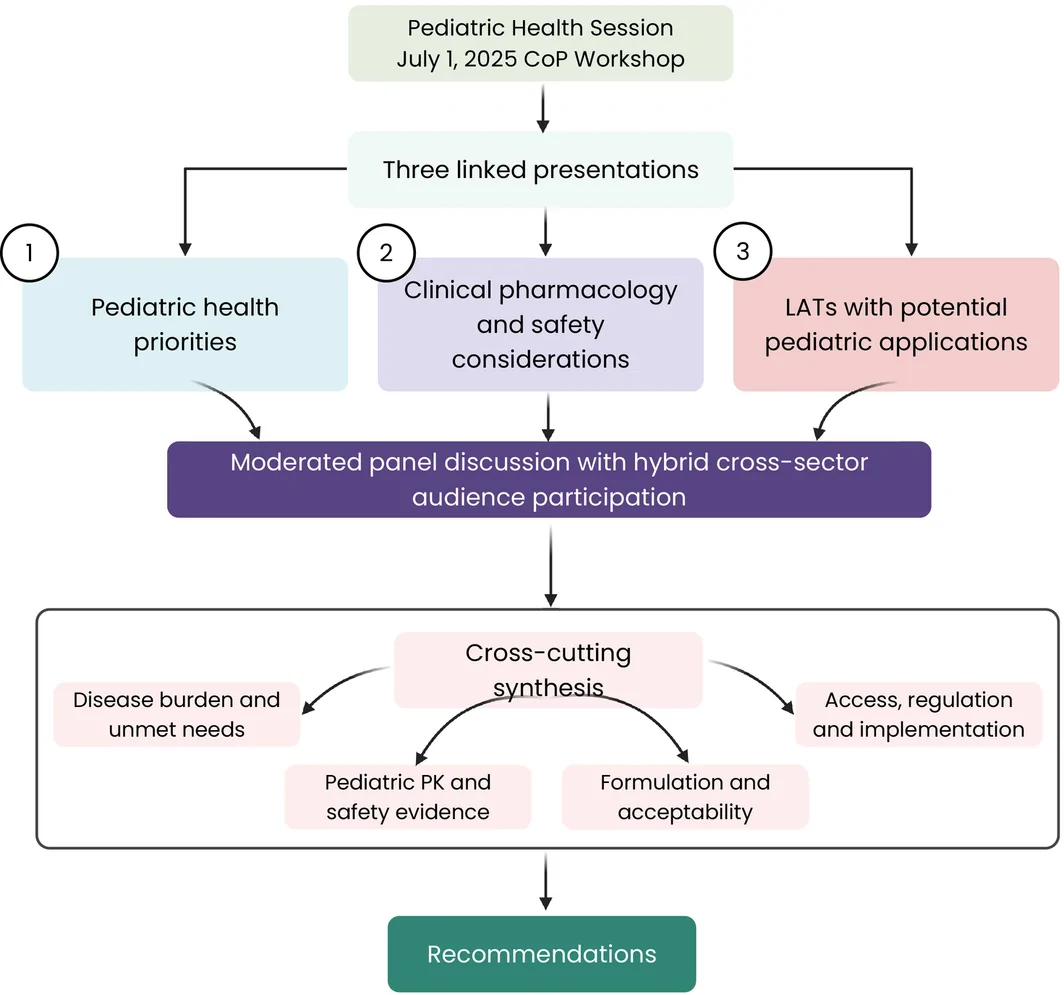
Framework for the pediatric health session of the Community of Practice workshop. The pediatric session comprised three linked presentations addressing pediatric health priorities, pharmacokinetic and safety considerations, and long‐acting therapeutic technologies for pediatric applications, followed by a moderated panel discussion and hybrid audience Q&A. Cross‐cutting synthesis of these discussions informed the panel recommendations on pediatric evidence generation, formulation and acceptability assessment, regulatory pathways, equitable access, and implementation. CoP, Community of Practice; LAT, long‐acting therapeutic; LMIC, low‐ and middle‐income country; PK, pharmacokinetic; TPP, target product profile.

## PEDIATRIC HEALTH PRIORITIES: IDENTIFYING WHERE THE NEED IS GREATEST

### Global trends in childhood mortality

According to the United Nations Inter‐Agency Group for Child Mortality Estimation (UN IGME), the global under‐five mortality rate fell by 52% between 1991 and 2022.^1^ Despite this progress, the pace of decline has slowed, and substantial geographic disparities persist, with the greatest burden of deaths occurring in Africa and South‐East Asia. Neonatal mortality, which accounts for nearly half of under‐five deaths, has declined more slowly than mortality among older infants and children. Interventions addressing maternal health, obstetric care, and early neonatal care are therefore essential to further reduce neonatal mortality.^4^ Beyond the neonatal period, mortality often reflects complex causal pathways involving malnutrition, HIV, malaria, congenital disorders, lower respiratory tract infections (LRTIs), diarrheal diseases, and sepsis. Infectious causes remain prominent: in the Child Health and Mortality Prevention Surveillance network, infection was present in the causal chain for 87% of in‐hospital deaths. Key pathogens included *Klebsiella pneumoniae*, *Plasmodium falciparum*, and *Streptococcus pneumoniae*.^5^

### Communicable diseases burden in children

Communicable diseases remain a leading cause of death among children aged 1 month to 9 years.^6^ According to the Global Burden of Disease Study (2019), children and adolescents account for 57% of the total communicable disease burden across all ages.^7^ Major contributors include enteric infections, LRTIs, malaria, tuberculosis, and HIV.

### Priority infectious diseases

#### Lower respiratory tract infections (LRTIs)

LRTIs, especially pneumonia, remain a leading cause of pediatric mortality worldwide. The introduction of vaccines against *Streptococcus pneumoniae* and *Haemophilus influenzae* has reduced the proportion of LRTIs caused by these infections, changing the pediatric respiratory infection landscape. A study of children hospitalized with severe pneumonia found that Respiratory Syncytial Virus (RSV) accounted for 31.1% of identified pathogens, with the highest burden among children younger than 1 year.^8^

RSV prevention strategies include maternal vaccination and long‐acting monoclonal antibodies (mAbs). Maternal vaccination has shown 81.8% effectiveness against severe RSV‐associated LRTI within 90 days of birth. Long‐acting mAbs demonstrated 82% effectiveness against RSV‐associated LRTI and 69.2% against all‐cause LRTI hospitalizations, with protection lasting approximately 6–9 months after administration.^9^, ^10^ RSV prevention also reduces antimicrobial use, with modeling and observational studies suggesting reductions up to 12.9% in antibiotic prescriptions among infants following RSV‐targeted interventions.^11^, ^12^, ^13^ In 2024, the WHO's Strategic Advisory Group of Experts recommended passive immunization as one of the strategies for RSV prevention in young infants. The dramatic reduction in RSV infections following the successful introduction of RSV long‐acting mAbs in several countries demonstrates the potential for long‐acting products to change the epidemiology and severity of an infectious disease. Affordability and access in LMICs remain critical priorities.^14^

#### Malaria

Malaria remains a major cause of pediatric morbidity and mortality, particularly among children under five in sub‐Saharan Africa. In 2023, an estimated 263 million malaria cases and 597,000 malaria deaths occurred globally, with countries including Nigeria, the Democratic Republic of the Congo, Niger, and Tanzania accounting for a substantial proportion of the burden.^15^ Addressing this challenge requires a combination of strategies, including vector control, immunization, early diagnosis and treatment, and chemoprevention for populations at highest risk, including children and pregnant women.^16^

Current oral chemoprevention strategies, including seasonal malaria chemoprevention and perennial malaria chemoprevention, can be limited by tolerability, adherence, duration of administration, and health‐system delivery challenges. Long‐acting innovations may help address some of these barriers. For example, MMV371, a LAI derivative of atovaquone, has been explored as a potential approach for up to 3 months of protection with a single intramuscular dose.^17^

#### Tuberculosis (TB)

According to WHO, TB affects approximately 1.25 million children annually, yet only 612,800 children, approximately 51%, are diagnosed and treated.^18^, ^19^ African countries contribute disproportionately to the global pediatric TB burden, with high incidence reported in countries including the Central African Republic, Namibia, Lesotho, and Gabon.^18^, ^19^ The WHO roadmap to end pediatric TB emphasizes early diagnosis, contact tracing, and preventive therapy.^20^

Several long‐acting formulations are being explored as potential tools to simplify TB prevention or treatment. Formulation development is planned or underway for several TB drugs, including rifapentine, rifabutin, isoniazid, delamanid, bedaquiline, TBAJ‐876, and TBAJ‐587, and a Phase 1 clinical trial of the LAI formulation of bedaquiline is planned.^21^ Shorter regimens, including HPZM, are also being tested in trials such as SMILE‐TB, which aim to shorten treatment duration while maintaining efficacy.^22^

#### HIV

Despite expanded prevention of mother‐to‐child transmission (PMTCT) programs, pediatric HIV remains a challenge.^23^ In 2023, approximately 120,000 new infections occurred in children. Treatment outcomes remain poorer than in adults: 63% of children know their HIV status, 57% are on ART, and 46% are virally suppressed. Innovations are therefore needed to improve postnatal prevention, treatment continuity, adherence, and long‐term viral suppression.^24^ For treatment of adolescents aged 12 years and older living with HIV‐1 infection, long‐acting cabotegravir and rilpivirine are approved for treatment and are under evaluation in children aged 2 to less than 12 years.^25^, ^26^ Current long‐acting HIV regimens still face challenges, including cold chain requirements, supply‐chain issues, and resistance risk in the context of interrupted therapy or prolonged subtherapeutic exposure.^27^ New drug delivery approaches, including dissolving microarray patches containing cabotegravir and rilpivirine nanosuspensions, are under investigation for pediatric HIV treatment.^28^

#### Syphilis

Congenital syphilis remains an important and resurgent global public‐health challenge. An estimated 661,000 cases occurred globally in 2016, and maternal syphilis remains a major driver of preventable fetal and neonatal morbidity and mortality. In South Africa, missed prevention opportunities have included inadequate treatment and diagnostic failure. Treatment depends on disease stage and clinical context and includes benzathine penicillin G or aqueous crystalline penicillin G.^29^, ^30^ Benzathine penicillin G is a long‐acting injectable formulation that remains central to syphilis management, but its use is associated with important implementation challenges, including injection pain and global supply constraints. In addition, treatment decisions depend on maternal history, serologic titers, physical examination findings, and disease stage, adding complexity to care. Alternative delivery strategies, including subcutaneous infusion approaches and alternative antibiotics, are being explored, although any replacement strategy would require robust evidence of efficacy, safety, and feasibility.^30^

### Other disease priorities

#### Childhood cancer

Childhood cancers, including leukemias, lymphomas, solid tumors, and sarcomas, remain an important cause of pediatric morbidity and mortality. The estimated incidence of primary cancer worldwide is 155.8 per million person‐years for individuals aged 0–19 and 140.6 per million person‐years for children aged 0–14. More than 200,000 children and adolescents are diagnosed with cancer each year, accounting for almost 1% of all cancers globally.^31^

Current pediatric cancer treatments include chemotherapy, immunotherapy, radiation therapy, and surgery, but many are associated with substantial short‐ and long‐term toxicities. Long‐acting or sustained‐release drug delivery systems, including polymer‐encapsulated nanoformulations and in situ forming gels, may offer opportunities to improve drug exposure profiles, reduce dosing burden, or limit systemic toxicity for selected agents.^31^ Pembrolizumab, administered intravenously every 3 weeks in some pediatric oncology indications, illustrates the relevance of extended‐interval biologic therapy in children, although it should not be described as a depot‐based long‐acting injectable.^32^

#### Growth hormone deficiency

Growth hormone deficiency is an uncommon pediatric endocrine disorder characterized by inadequate growth hormone secretion due to congenital or acquired causes. Standard treatment has traditionally involved daily recombinant human growth hormone injections.^33^ Long‐acting growth hormone formulations are now being developed or introduced to reduce injection burden and potentially improve adherence and clinical outcomes.^34^ These products illustrate how long‐acting technologies may address pediatric needs beyond infectious diseases, particularly where chronic therapy requires repeated administration over many years.

## PHARMACOKINETIC AND SAFETY CONSIDERATIONS FOR LONG‐ACTING THERAPEUTICS

The pediatric disease priorities outlined above highlight both the scale of unmet clinical need and the limitations of existing treatment and prevention strategies. In this context, LATs offer the potential to maintain drug exposure over extended periods, reduce dosing frequency, and improve adherence. However, the PK and tolerability of LATs in children cannot be directly inferred from either conventional formulations or adult data. Developmental changes in body composition, organ maturation, drug disposition, and route‐specific physiology can all influence the absorption, distribution, metabolism, excretion, and tolerability of long‐acting products in pediatric populations. A clear understanding of these factors is therefore essential for the rational development, dose selection, and implementation of LATs in children.^35^, ^36^

### Unique clinical pharmacology issues with long‐acting agents

**Figure 2:**
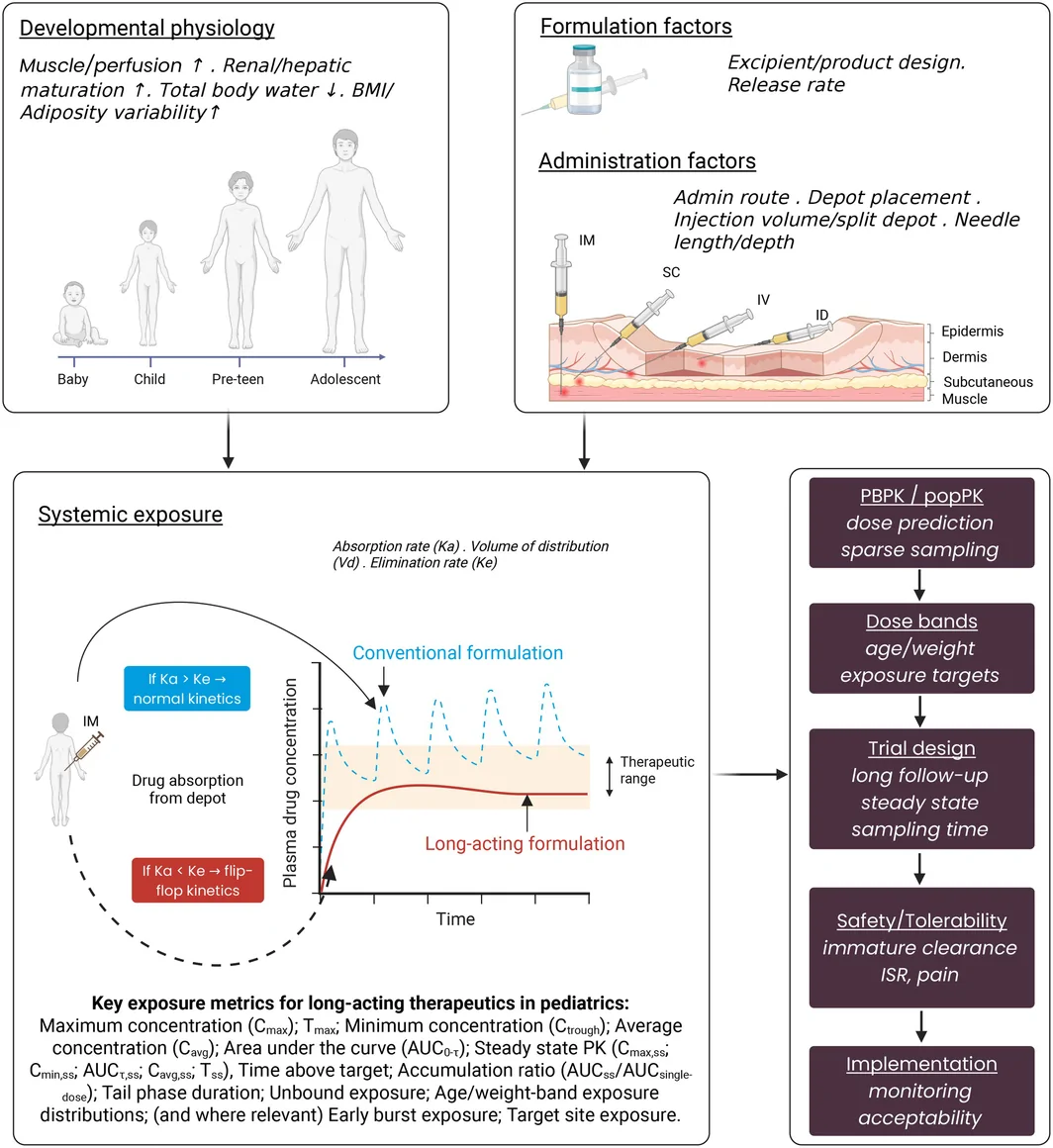
Developmental and formulation determinants of long‐acting therapeutic exposure in pediatric populations. Age‐dependent maturation and product‐ or administration‐related factors influence Ka, Ke, and Vd, thereby shaping systemic exposure, steady‐state attainment, and the pharmacokinetic tail. These exposure characteristics inform model‐informed dose selection, safety and tolerability assessment, discontinuation planning, and implementation. IM, intramuscular; SC, subcutaneous; IV, intravenous; ID, intradermal; Ka, absorption rate constant; Ke, elimination rate constant; Vd, volume of distribution; ISR, injection‐site reaction.

Long‐acting products present distinct clinical pharmacology challenges in pediatric populations because both the route of administration and developmental physiology can influence the rate and extent of drug exposure. For depot‐based long‐acting formulations, age‐related differences in tissue composition, local blood flow, and injection‐site anatomy can alter absorption kinetics (**Figure** **2**). In parallel, maturation of hepatic enzymes and renal function may affect systemic disposition once the drug has entered the circulation. These interacting factors are particularly important in neonates, infants, and young children, in whom physiological systems are still developing and inter‐individual variability is often greater than in older children and adults.^37^, ^38^, ^39^

Renal ontogeny is especially relevant to dose selection and PK interpretation for pediatric LATs. Renal clearance comprises glomerular filtration, tubular secretion, and tubular reabsorption, all of which mature postnatally. In full‐term neonates, glomerular filtration rate (GFR) is low at birth, typically around 10–20 mL/min/1.73 m^2^, but rises rapidly during the first months of life, reaching near‐adult values by approximately 3–5 months. Tubular secretion and reabsorption, by contrast, mature more gradually and may not reach full functional capacity until around 2 years of age. Hepatic metabolic capacity is also developmentally regulated. Drug‐metabolizing enzymes, including cytochrome P450 (CYP) isoforms such as CYP3A, CYP2C19, and CYP2D6, and uridine 5′‐diphospho‐glucuronosyltransferases (UGTs), generally have reduced activity at birth. Although activity increases rapidly during infancy, maturation may extend beyond the first year of life and differs by enzyme isoform. Consequently, for LATs whose released parent drug or metabolites undergo hepatic metabolism, knowledge of the relevant metabolic pathways is essential for predicting age‐dependent clearance, exposure, accumulation risk, and dosing intervals. Even where absorption from a depot formulation is rate‐limiting, immature drug clearance may still influence systemic exposure to the released parent drug or metabolites, particularly during early post‐dose periods, loading phases, or discontinuation. Consequently, maturation of drug elimination processes should be considered alongside absorption kinetics when selecting pediatric doses and dosing intervals for long‐acting agents.^39^, ^40^

For many depot‐based long‐acting small molecules, the terminal phase of the plasma concentration–time profile may reflect absorption rather than elimination when the absorption rate constant (K_a_) is lower than the elimination rate constant (K_e_), a phenomenon commonly described as flip‐flop kinetics. Under these conditions, prolonged release from the depot, rather than systemic clearance, determines the apparent terminal slope (λz) and contributes to the peak‐to‐trough fluctuation within a dosing interval and to delayed attainment of steady state. Adult clinical data for long‐acting cabotegravir illustrate this principle, with an extended decline phase following cessation of dosing attributable to prolonged absorption from the intramuscular depot.^41^ This has important implications for pediatric development, because exposure cannot be interpreted solely through conventional assumptions about clearance and half‐life.^38^, ^42^

These complexities strengthen the case for model‐informed pediatric drug development. Because long‐acting formulations may take weeks or months to approach steady‐state conditions, early exposure targets must often be predicted rather than observed directly in the first instance. PBPK and population PK approaches are therefore valuable tools for pediatric dose selection (**Figure** **2**), particularly when adult data exist but direct pediatric sampling is limited.^42^, ^43^ Such models can incorporate age‐dependent changes in total body water, muscle mass, adiposity, organ maturation, and clearance pathways.^44^ In infants, for example, total body water is proportionally higher than in adults, and muscle mass constitutes a smaller fraction of body weight, both of which may influence depot disposition and systemic exposure after intramuscular administration.^38^, ^44^ Published PBPK work has shown that age‐ and weight‐based dose‐escalation strategies can support the selection of cabotegravir and rilpivirine regimens predicted to achieve target trough concentrations across pediatric age groups.^45^

Clinical study design for pediatric LATs is also challenging. Single‐dose studies may require prolonged follow‐up to characterize exposure adequately and estimate AUC(0–∞), while multidose studies may require extended observation before steady‐state AUC(0–τ) can be assessed.^46^ This is particularly burdensome in pediatric populations, where repeated blood sampling, long clinic attendance, and delayed interpretability of results can complicate recruitment and retention. For these reasons, pediatric LAT development is likely to depend heavily on integrated strategies that combine adult data, sparse pediatric sampling, model‐based predictions, and age‐appropriate dose‐banding approaches.^37^, ^43^, ^45^, ^46^

### PK tailing, resistance risk, and implications for anti‐infectives

A further consideration for long‐acting anti‐infectives is the pharmacokinetic “tail” that follows the final administered dose. Adult studies of long‐acting cabotegravir have demonstrated a prolonged decline phase during which low but measurable drug concentrations persist for extended periods.^41^ In the context of anti‐infective therapy or prophylaxis, this may be clinically important because prolonged subtherapeutic exposure can increase the risk of resistance selection, particularly where treatment is interrupted, adherence is incomplete, or active infection is present.^47^ Although analogous pediatric PK‐tail data remain limited, the issue is highly relevant to pediatric long‐acting development and should be considered in dose selection, monitoring strategies, and discontinuation planning. Current efforts to address this evidence gap include an ongoing trial of long‐acting lenacapavir (PURPOSE 365; NCT07047716), which is evaluating PK, safety, and the tail phase in adolescents aged 16 years and older receiving HIV pre‐exposure prophylaxis (PrEP).^48^

### Factors affecting absorption in flip‐flop kinetics

In pediatric long‐acting injectables, age‐related differences in skeletal muscle mass, adipose tissue thickness, body water composition, and regional perfusion can influence depot formation and the rate of absorption after intramuscular (IM) or subcutaneous (SC) administration.^39^, ^44^ In neonates and young infants, low muscle mass and reduced muscular perfusion may delay and increase variability in IM absorption.^39^, ^49^ In older children and adolescents, variability in body composition becomes increasingly relevant, particularly for lipophilic agents and for products in which absorption from the depot is highly sensitive to injection depth and local tissue characteristics.^38^, ^44^, ^50^, ^51^, ^52^

Adult population PK data for long‐acting cabotegravir provide useful mechanistic insights that are likely to be relevant in pediatrics. Han et al. reported that shorter needle length was associated with slower absorption, while split injection techniques increased Ka, plausibly because two smaller depots provide a greater effective surface area for absorption than one larger‐volume depot.^40^ The same analysis also suggested that higher body mass index (BMI) was associated with lower Ka and reduced exposure, likely reflecting greater subcutaneous fat thickness and a reduced likelihood of consistent IM delivery into muscle.^40^ Extrapolated to pediatric practice, these findings support careful attention to needle length, injection technique, and body habitus. Needle length and injection technique should therefore be selected according to age, body habitus, injection site, and tissue depth to support reliable intramuscular administration while avoiding inappropriate depot placement.^53^

Beyond absorption, developmental changes in body composition also influence drug distribution. Relative total body water, fat fraction, and lean body mass vary substantially across pediatric age groups and may alter the apparent volume of distribution for both hydrophilic and lipophilic agents.^44^, ^50^, ^51^, ^52^ In overweight or obese children, these shifts may be particularly relevant and can contribute to exposure variability for long‐acting products. Collectively, these developmental and formulation‐related factors influence the balance between K_a_ and K_e_ and therefore the likelihood and extent of flip‐flop kinetics.^42^ Flip‐flop kinetics of long‐acting drugs are characterized by K_a_ < K_e_; consequently, the apparent terminal half‐life primarily reflects absorption rather than elimination (**Figure** **2**). A robust understanding of factors affecting absorption is therefore essential when interpreting terminal half‐life, predicting steady‐state attainment, and designing pediatric dosing regimens.^37^, ^38^, ^39^, ^49^

### Pharmacokinetics and injection‐site tolerability of long‐acting injectable therapies

Tolerability at the site of administration is a critical determinant of the feasibility and acceptability of pediatric LATs. Experience with long‐acting cabotegravir and rilpivirine suggests that exposures in children and adolescent comparable to those in adults can be achieved with appropriately selected age‐ or weight‐based regimens.^46^, ^54^ In the IMPAACT/MOCHA program, long‐acting cabotegravir and rilpivirine administered to virologically suppressed adolescents demonstrated PK profiles broadly similar to adults, with injection‐site reactions (ISRs) generally mild to moderate in severity.^46^, ^54^ However, even when clinically manageable, ISRs remain highly relevant in children and adolescents because pain, anxiety, and aversion to repeated procedures may affect treatment uptake, adherence, and overall acceptability.^54^, ^55^ Accordingly, pediatric LAT studies should assess not only the frequency and severity of injection‐site reactions but also age‐appropriate acceptability, child/caregiver preference, willingness to continue treatment, and the practical burden of repeated administration.

It is also important to distinguish between depot‐based long‐acting injectables and therapies given at extended dosing intervals for other pharmacological reasons. For example, pembrolizumab administered intravenously every three weeks in pediatric oncology populations has shown exposures comparable to adults and no dose‐limiting toxicities in early‐phase evaluation.^32^ Although this is not a depot‐based long‐acting injectable in the same sense as IM or SC formulations, it illustrates a broader point relevant to pediatric development: where exposure matching to adults is the primary objective, pediatric PK evaluation must still account for developmental differences in disposition and safety.^32^, ^56^

### Developmental pharmacokinetics and dosing principles for pediatric long‐acting formulations

Overall, pediatric dose selection for LATs should aim to achieve exposure and safety profiles appropriately comparable to those established in adults, while explicitly accounting for developmental differences in absorption, distribution, metabolism, excretion, and tolerability.^56^, ^57^ This is especially important in neonates, infants, and younger children, where immature clearance pathways and route‐specific physiological differences may increase the risk of underexposure, delayed attainment of target concentrations, or drug accumulation.^39^, ^56^, ^58^ Accordingly, long‐acting formulations should not simply be down‐scaled from adult regimens on a milligram‐per‐kilogram basis without consideration of developmental pharmacology.^37^, ^39^, ^56^

Population PK and PBPK modeling are therefore likely to play central roles in pediatric LAT development, including the selection of dose bands, dosing intervals, and sampling schedules.^43^, ^45^, ^57^ These approaches are particularly helpful where conventional richly sampled trials are impractical, and where extrapolation from adult efficacy data may be justified provided that pediatric exposure, safety, and tolerability are adequately characterized.^56^, ^57^ Experience with cabotegravir/rilpivirine further suggests that pediatric development strategies may need to incorporate formulation‐specific initiation approaches, including oral lead‐in or alternative loading strategies where appropriate, although such approaches should be considered on a product‐by‐product basis rather than assumed to be universally necessary.^55^, ^59^

For pediatric long‐acting therapeutics, exposure assessment should extend beyond conventional *C*_max_ and AUC metrics to include parameters that describe sustained pharmacological coverage, depot‐release behavior, accumulation, and washout. Relevant measures include *C*_max_, *T*_max_, AUC over the dosing interval, average concentration, trough concentration, time above the pharmacodynamic target, early burst exposure, accumulation ratio, terminal tail‐phase duration, and, where relevant, unbound or target‐site concentrations stratified by age, weight, and developmental physiology (**Figure** **2**). Taken together, these considerations support an integrated pediatric development strategy in which developmental pharmacology, formulation science, age‐appropriate tolerability assessment, and model‐informed dose optimization are incorporated from the earliest stages.^35^, ^43^, ^45^, ^57^

## LAT TECHNOLOGIES FOR PEDIATRIC HEALTH PRIORITIES

LAT technologies comprise a diverse group of drug delivery and half‐life extension platforms designed to sustain therapeutic exposure over extended periods, reduce dosing frequency, and lessen treatment burden.^60^ These attributes are particularly relevant to pediatric health priorities, where frequent dosing, palatability, caregiver administration, access to healthcare facilities, and adherence can all limit treatment effectiveness. However, pediatric use of LAT technologies remains limited, and children frequently receive extrapolated or off‐label regimens because of insufficient age‐specific pharmacokinetic, safety, acceptability, and formulation data.^35^, ^36^

LAT platform technologies can be broadly grouped according to their route and mechanism of prolonged exposure. Injectable depot systems include aqueous suspensions, oil‐based depots, in situ forming systems, and polymeric microspheres such as poly(lactic‐co‐glycolic acid) formulations.^60^ Other approaches include subdermal implants, transdermal or intradermal systems such as microneedles, long‐acting oral or gastroretentive systems, and biologic half‐life extension technologies.^60^, ^61^, ^62^ These platforms can be used to deliver a wide range of therapeutic modalities, including small molecules, peptides, proteins, monoclonal antibodies, and other biologics.^60^, ^61^ Their suitability for children depends not only on the active drug and duration of release, but also on route of administration, excipient burden, device or depot size, reversibility, tolerability, and feasibility of use across pediatric age groups.

### General considerations for the development of LAT technologies in pediatrics

Several formulation and implementation considerations are especially important before LAT technologies are used widely in pediatric populations. Although parenteral administration is routine in pediatric care, depot‐forming or repeated injections may be limited by injection‐site pain, anxiety, acceptability, sterility requirements, local tissue reactions, and the need for trained personnel.^63^ These issues may be particularly relevant for children requiring chronic treatment or repeated prophylaxis. In addition, some LA technologies, including oil depots and in situ forming systems, may present formulation‐specific challenges related to physical stability, chemical stability, injection volume, depot formation, and local tissue tolerability in developing pediatric tissues.^64^

Route of administration should therefore be selected with age, anatomy, formulation properties, and health‐system feasibility in mind. Intravenous administration allows controlled systemic delivery but requires venous access, trained staff, monitoring, and infection‐control infrastructure, making it unsuitable for many depot‐based LAT technologies. Intramuscular (IM) administration can support depot formation and prolonged release, but low muscle mass and reduced muscle perfusion in preterm neonates, neonates, and young infants may limit reliability and acceptability.^53^, ^56^ Subcutaneous delivery may be less invasive and more feasible for some products, but it remains constrained by injection volume, local tolerability, tissue thickness, and absorption variability. Transdermal, intradermal, implantable, and oral long‐acting platforms may reduce the burden of repeated injections, but each introduces age‐specific design constraints, including skin maturation, device size, swallowing ability, removability, and caregiver or healthcare‐provider usability.

A critical formulation consideration for pediatric LAT technologies is the potential for initial burst release. Initial burst refers to the rapid release of a fraction of the drug soon after administration, before the intended sustained‐release phase is established.^65^ In children, this may be particularly important because smaller body size, immature clearance pathways, and narrower safety margins may increase the risk of transient supratherapeutic exposure if early release is excessive.^66^ For pediatric LAT technology development, initial burst should therefore be evaluated alongside longer‐term exposure, local tolerability, and dose proportionality.

### Physiological considerations for the development of LAT technologies in pediatrics

Pediatric physiology changes substantially from birth through adolescence, and these changes should inform LAT platform technology selection. Age‐dependent differences in body composition, tissue perfusion, skin structure, gastrointestinal function, renal maturation, and hepatic enzyme activity can influence formulation performance, drug release, absorption, distribution, and clearance.^67^ These factors are most pronounced in neonates and infants, but they remain relevant across childhood because body size, adiposity, muscle mass, and organ function continue to evolve.

For oral LATs, developmental differences in gastric pH, intestinal transit, feeding patterns, swallowing ability, and gastrointestinal maturation may affect drug release and absorption. For transdermal and intradermal systems, immature skin structure and a higher body surface area‐to‐weight ratio in neonates and infants may increase percutaneous absorption and systemic exposure.^68^, ^69^ Conversely, for intramuscular LATs, low muscle mass and reduced muscle blood flow in neonates and young infants may delay or increase variability in absorption.^49^ These considerations reinforce the need for pediatric‐specific formulation evaluation rather than direct extrapolation from adult LAT performance.

Overall, pediatric LAT development should integrate formulation science with developmental pharmacology from the earliest stages. Adult PK and safety data are informative but are unlikely to be sufficient on their own for pediatric use, particularly when the formulation, route, depot behavior, or excipient exposure differs across age groups.^70^ Platform selection should therefore consider the intended pediatric population, route feasibility, dose flexibility, excipient safety, local tolerability, caregiver and child acceptability, and the ability to generate robust age‐appropriate pharmacokinetic and safety evidence.

Among these design considerations, excipient selection is particularly important for pediatric LAT technologies because long‐acting systems may require higher or more persistent excipient exposure than conventional formulations.

### Excipient safety and developmental toxicology considerations in pediatric LAT technologies

LAT technologies often require specialized excipients to enable drug solubilization, encapsulation, depot formation, controlled release, stabilization, or local tissue retention. The type and quantity of excipients may therefore differ substantially across LAT platform technologies, including suspensions, in situ forming depots, polymeric systems, lipid‐based matrices, implants, microneedles, and long‐acting oral systems.^60^ In pediatric populations, excipient selection requires particular caution because developmental differences in renal and hepatic clearance, body composition, skin and tissue permeability, and metabolic capacity may alter excipient disposition and toxicity risk. Although some excipients used in pharmaceutical formulations may be listed as Generally Recognized as Safe (GRAS) or may appear in the United States Food and Drug Administration inactive ingredient database, these designations should not be interpreted as universal evidence of safety for all pediatric populations, routes of administration, doses, or durations of exposure.^71^ This distinction is especially important for LATs, where excipients may be present at high local concentrations, remain at the administration site for prolonged periods, or contribute to sustained systemic exposure. Accordingly, pediatric LAT development should consider not only the active pharmaceutical ingredient but also the total excipient burden, route of administration, age group, dosing interval, and expected duration of exposure.^72^

Potential systemic toxicity risks and rational excipient selection: Synthetic and semi‐synthetic polymers, surfactants, solvents, cosolvents, preservatives, and depot‐forming agents may all contribute to the performance of LAT technologies, but their safety profiles may differ across pediatric age groups. For example, excipients such as polyethylene glycol‐containing compounds, polysorbates, propylene glycol, ethanol, and benzyl alcohol may raise age‐, dose‐, and route‐dependent concerns, particularly in neonates and young infants with immature metabolic and renal clearance pathways.^73^ In particular, propylene glycol (PG) has documented neonatal toxicity at excessive exposures, including hyperosmolality, renal and hepatic dysfunction, cardiotoxicity, CNS toxicity, and respiratory depression.^74^ Acceptable propylene glycol exposure is age dependent and should be assessed against current regulatory guidance, with particular caution in neonates and young infants because of their limited metabolic and renal clearance capacity. Benzyl alcohol and ethanol also require caution in newborns and infants because of limited metabolic capacity and the potential for systemic toxicity.^73^

The selection of excipients for pediatric LATs should therefore be guided by a structured risk assessment rather than by adult formulation precedent alone. Key considerations include prior pediatric use, maximum daily exposure, local tissue tolerability, systemic toxicokinetics, potential accumulation after repeated dosing, degradation products, immunogenicity, and compatibility with the intended route of administration. Where possible, formulators should prioritize excipients with established pediatric safety experience at relevant doses and routes, minimize unnecessary excipient burden, and generate age‐appropriate toxicology or toxicokinetic data when evidence is limited. Candidate alternatives such as microcrystalline cellulose, starches, selected polysaccharides, PEG‐based excipients, or preservatives may be appropriate in specific contexts, but their suitability should be evaluated on a case‐by‐case basis according to formulation type, administration route, dose, and pediatric age group.^73^

### Route of administration for pediatric LAT technologies: Advantages and limitations

Building on the physiological and formulation considerations outlined above, route selection for pediatric LATs should be viewed as a product‐development decision rather than a simple choice of delivery site. The optimal route will depend on the intended pediatric age group, required duration of exposure, dose and injection volume, formulation viscosity, excipient burden, local tissue tolerability, reversibility or removability, and feasibility of administration in routine care settings.^60^, ^64^

IM and SC routes remain the most established approach for many LATs, particularly where sustained release depends on depot formation. However, pediatric suitability varies according to age, anatomy, and product design. IM delivery may be appropriate for older children and adolescents when depot placement can be achieved reliably, whereas SC delivery may be preferable for products requiring less invasive administration or compatibility with self‐ or caregiver‐assisted delivery in older pediatric groups.^53^, ^56^, ^64^ In both cases, acceptability, pain mitigation, injection‐site reactions, and maximum feasible dose volume should be evaluated early in development rather than treated as late‐stage implementation considerations.^75^ Non‐parenteral and minimally invasive approaches may offer advantages where repeated injections are unacceptable or impractical. Transdermal, intradermal, microneedle, implantable, and long‐acting oral systems may reduce procedure‐related burden, but each introduces distinct constraints, including dose‐loading capacity, adhesion or device retention, swallowing ability, insertion or removal requirements, local tissue response, and consistency of drug release. For implantable or in situ forming systems, reversibility, depot persistence, initial burst release, and excipient safety are particularly important pediatric considerations.^69^, ^71^

Overall, no single route is optimal across all pediatric age groups, indications, or LAT platform technologies. IM and SC administration currently represent the most mature routes for many long‐acting products, while transdermal, intradermal, implantable, and oral long‐acting systems may be preferable where they improve acceptability, reduce healthcare contact, or better match the intended duration of therapy.^60^, ^64^, ^69^ Route selection should therefore be tailored to the disease context, therapeutic index, developmental stage, formulation characteristics, and health‐system setting.

### Promising LAT platform technologies for pediatric applications

Against this background, we highlight selected LAT platform technologies with potential relevance to pediatric applications, including transient conjugated prodrug technologies, long‐acting hybrid Fc fusion systems, protein/peptide–Fc fragment conjugates, in situ forming lipid‐based nanocrystal gels, microneedle patches, and long‐acting gastric‐resistant oral capsules (**Table** **1**). These examples illustrate how different platform technologies may address pediatric needs by reducing dosing frequency, sustaining therapeutic exposure, improving acceptability, or enabling alternative routes of administration.

**Table 1:** Example LAT platform technologies with the route of administration, potential applications, frequency of administration, and pediatric suitability

Technology	Route of administration	Potential applications	Frequency	Pediatric suitability
Transient Conjugated Prodrug	Subcutaneous	Pediatric Growth Hormone Deficiency, Hypoparathyroidism	Once weekly	Approved for children; excipients with established use in the approved pediatric product; minimal injection‐site pain
Hybrid Fc Fusion Technology	Subcutaneous	Pediatric Growth Hormone Deficiency	Weekly/monthly	In phase III trials, excipient safety needs confirmation
Protein/Peptide–Fc Fragment Conjugation	Subcutaneous	Congenital hyperinsulinism	Once weekly	Phase 2 clinical evaluation includes children aged ≥2 years
In Situ Lipid‐Based Nanocrystal Gel	Subcutaneous	Genetic obesity disorders	Weekly	In a phase III clinical trial in children aged ≥6 years
Microneedle Patch	Transdermal/Intradermal	HIV	Weekly/monthly	High potential for minimally invasive delivery; no approved clinical product to date
Long‐Acting Gastric‐Resistant Capsule	Oral	HIV, Malaria	Weekly/monthly	Capsule size limits use in young children; suitable for adolescents and children

For pediatric development, the suitability of each LAT platform technology should be assessed in relation to the intended indication, therapeutic index, target age group, required duration of exposure, and feasibility of real‐world use. Key platform‐specific considerations include dose flexibility, reliability of release kinetics, acceptable local and systemic tolerability, manageable excipient burden, and—where relevant—control of initial burst and pharmacokinetic tail.^60^, ^64^, ^69^, ^71^ For anti‐infective agents, the pharmacokinetic tail warrants particular attention because prolonged subtherapeutic exposure after discontinuation may increase the risk of resistance. No single LAT platform technology will be optimal across all pediatric populations or indications; therefore, technology selection should be matched to the disease context, developmental stage, formulation characteristics, and implementation setting. Additional information on long‐acting technologies, patents, and licensing landscapes can be obtained from LAPaL.^61^

#### Transient conjugated prodrug technology

**Figure 3:**
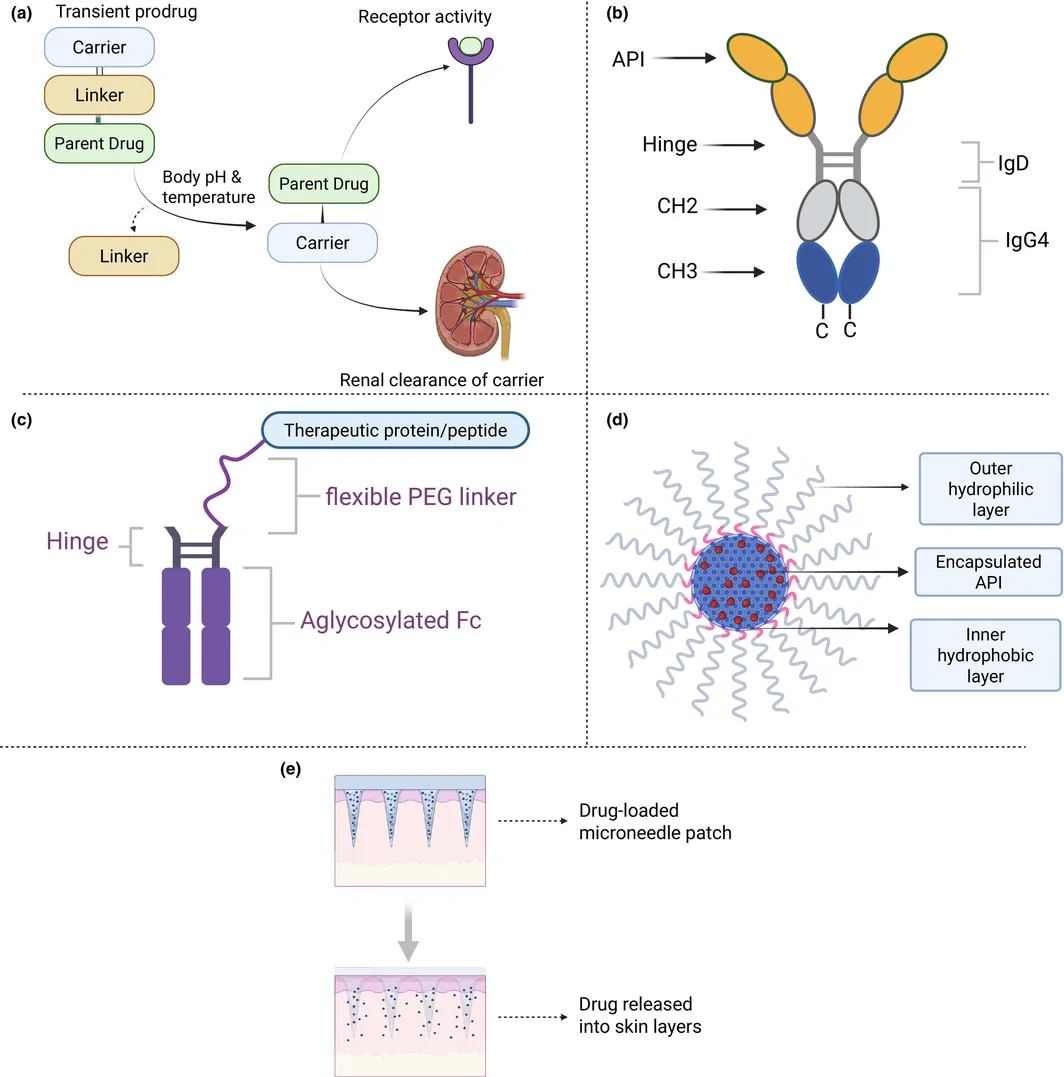
Representative long‐acting therapeutic technology platforms with potential pediatric applications. (**a**) Transient prodrug technology. The parent drug is conjugated to a carrier through a reversible linker to form a transient prodrug complex. Under physiological conditions, linker cleavage releases the active parent drug in a controlled manner, supporting sustained exposure, while the carrier is subsequently cleared. (**b**) Hybrid Fc fusion technology. The active pharmaceutical ingredient (API) is fused to an engineered hybrid Fc domain designed to prolong systemic exposure by improving molecular stability and reducing clearance. (**c**) Protein/peptide–Fc fragment conjugation technology. A therapeutic protein or peptide is conjugated to aglycosylated Fc‐derived fragments through a flexible polyethylene glycol (PEG) linker, increasing molecular size and prolonging systemic exposure. (**d**) In situ forming lipid‐based nanocrystal gel. Following administration, self‐assembling lipids form a structured depot with hydrophilic and hydrophobic domains that can incorporate the API and enable controlled release over time. (**e**) Dissolving polymer‐based microneedle patch. Drug‐loaded microneedles penetrate the superficial skin layers and dissolve following application, releasing the drug for local or systemic absorption.

Transient conjugated prodrug technology converts an API into a reversibly conjugated prodrug through attachment to a carrier via a cleavable linker (**Figure** **3** **a**).^76^ Under physiological conditions, linker cleavage releases the active drug in a controlled manner, thereby enabling sustained exposure. This approach can be applied to selected small molecules, peptides, and proteins, and may support high drug loading depending on the carrier‐linker system and physicochemical properties of the API.^76^

The platform can use soluble carriers, such as polyethylene glycol, or insoluble carriers, such as lipid‐based systems, depending on the desired stability, release profile, and route of administration. Carrier conjugation can reduce premature receptor interaction, delay renal clearance, and protect susceptible APIs from enzymatic degradation. Reversible linker chemistries include aromatic cyclic imide, 2,5‐diketopiperazine, carbamate, bicin, 2‐((2‐aminoethyl)amino)acetic acid, and pyroglutamate linkers.^77^ Pediatric applications are most advanced in endocrinology, where lonapegsomatropin provides a long‐acting growth hormone option for children with growth hormone deficiency.^78^ The same transient conjugation platform has also supported other endocrine products, including palopegteriparatide, a prodrug approved for adult hypoparathyroidism, illustrating the broader applicability of the carrier‐linker approach beyond pediatric growth hormone deficiency.^79^ However, pediatric use of each product should be assessed independently, with attention to age‐appropriate dosing, excipient exposure, injection burden, and evidence of sustained efficacy and safety across pediatric subgroups.

#### Long‐acting hybrid Fc fusion technology

Hybrid Fc fusion technologies are designed to extend the systemic half‐life of therapeutic proteins by exploiting Fc‐mediated recycling pathways and improved molecular stability. These platforms typically combine a therapeutic protein or peptide with an engineered Fc domain and may incorporate additional protein components to optimize stability, exposure, or biological activity (**Figure** **3** **b**).^80^ Rather than functioning primarily through depot absorption, Fc fusion approaches prolong exposure mainly through reduced clearance and extended systemic persistence.

In pediatric development, these technologies are particularly relevant for conditions requiring chronic replacement or repeated biologic therapy, such as growth hormone deficiency. Eftansomatropin alfa, a long‐acting human growth hormone product, is currently being evaluated in phase III clinical development for pediatric growth hormone deficiency.^81^ Key pediatric considerations include immunogenicity, exposure matching across body size and developmental stages, dose flexibility, injection frequency, and long‐term safety monitoring.

#### Protein/peptide–Fc fragment conjugation technology

Protein/peptide–Fc fragment conjugation is a half‐life extension strategy in which a therapeutic protein or peptide is linked to Fc‐derived fragments, often through a flexible linker such as polyethylene glycol, to increase molecular size, reduce clearance, and prolong systemic exposure (**Figure** **3** **c**). In some platforms, the API is conjugated to two aglycosylated monomeric Fc fragments via a flexible PEG linker, which can limit receptor interaction and delay elimination.^82^ Unlike depot‐forming long‐acting injectables, this approach primarily extends exposure by modifying systemic disposition rather than by controlling absorption from an injection‐site depot.

This platform may be applicable to a range of protein and peptide therapeutics, including insulin analogues, growth hormone products, glucagon‐like peptide‐1 receptor agonists, interleukins, epidermal growth factors, and selected small molecules where Fc‐based conjugation is feasible.^82^ A pediatric‐relevant example is efpegerglucagon, a glucagon‐IgG4 conjugate developed for once‐weekly subcutaneous administration that has received US FDA orphan drug designation for congenital hyperinsulinism.^83^ Its suitability for individual pediatric age groups requires clinical evaluation. For pediatric development, key considerations include dose flexibility across age and weight bands, immunogenicity assessment, injection burden, local tolerability, and the long‐term consequences of sustained pharmacological pathway modulation.

#### In situ forming lipid‐based nanocrystal gel technology

In situ forming lipid‐based nanocrystal gel systems use self‐assembling lipids that form structured depots after administration, commonly by subcutaneous injection. Lipids such as neutral diacyl lipids, tocopherol, cardiolipin, phosphatidylinositol, phosphatidylethanolamine, and phospholipids can form three‐dimensional non‐lamellar lipid crystalline structures capable of incorporating hydrophobic, hydrophilic, and amphiphilic molecules (**Figure** **3** **d**).^84^ These depots can gradually dissolve or reorganize in tissue, releasing the therapeutic agent over time.

A weekly setmelanotide in situ gel is being studied for rare genetic disorders of obesity, including Bardet‐Biedl syndrome, in children aged 6 years and older.^85^ For pediatric use, key considerations include injection volume, local tissue response, initial burst release, depot persistence, reversibility, and excipient safety across age groups.

#### Microneedle patch

Microneedle patches are minimally invasive platforms designed for transdermal or intradermal delivery. Major types include dissolving, coated, and hydrogel‐forming microneedles (**Figure** **3** **e**).^86^ Microneedles are typically 0.1–1 mm in length and may be fabricated from polymers such as polyvinyl alcohol, polyvinylpyrrolidone, or other low‐molecular‐weight polymer systems, depending on the intended drug, release profile, and mechanical requirements.^86^, ^87^

For pediatrics, microneedle systems are attractive because they may reduce needle‐associated pain, avoid sharps injury, and potentially enable caregiver‐ or self‐administration in selected age groups. However, pediatric translation remains limited by dose‐loading capacity, skin maturation, adhesion, application reliability, local skin tolerability, caregiver usability, manufacturing scale‐up, and regulatory requirements. At present, clinical implementation of microneedle‐based drug products remains limited, and further pediatric‐specific evaluation is needed to inform potential implementation.^88^

#### Long‐acting gastric‐resistant oral capsule technology

Long‐acting gastric‐resistant oral capsule systems are designed to reside in the stomach and release drug over an extended period. Unlike conventional oral capsules that release drug over a short time frame, these systems may use a stellate or expandable structure composed of drug‐loaded arms connected to a flexible core, enabling gastric retention and sustained release.^89^ The arms are folded within the capsule before administration and expand after reaching the stomach.

This approach has been explored for small molecules such as cabotegravir, ivermectin, memantine, and buprenorphine.^90^, ^91^, ^92^ Its pediatric applicability is likely to be age‐dependent because capsule dimensions, swallowing ability, gastrointestinal anatomy, gastric residence time, and safety of prolonged gastric retention may limit use in younger children.^92^ Therefore, long‐acting oral systems may be more feasible for adolescents or older children unless smaller, age‐appropriate formats can be developed.

## PITFALLS AND PROMISES OF PEDIATRIC LAT TECHNOLOGIES

Although pediatric LATs include injectable, implantable, transdermal, intradermal, oral, and biologic half‐life extension platforms, LAIs currently represent the most mature and clinically visible modality. Therefore, their promises and limitations provide an important example of the broader opportunities and challenges facing pediatric LAT development.

LAIs offer several potential advantages for pediatric care, including reduced dosing frequency, sustained therapeutic or prophylactic exposure, fewer missed doses, reduced caregiver administration burden, and fewer healthcare contacts for conditions that otherwise require frequent dosing. These benefits may be particularly important for chronic diseases, infection prevention, and settings where adherence to daily oral therapy is difficult. However, the advantages of LAIs must be balanced against pediatric‐specific challenges related to administration, tolerability, acceptability, and implementation.

Injection‐site pain, local reactions, dose volume, product viscosity, needle length, and the need for trained administration may limit acceptability, especially in younger children or those requiring repeated injections.^35^, ^93^ Needle‐related fear, anxiety, and anticipated pain may further affect willingness to initiate or continue treatment.^94^ Pediatric experience with long‐acting antiretrovirals suggests that injection‐site reactions are often mild to moderate but remain clinically relevant. For example, an IMPAACT study reported injection‐site pain in 30% (9/30) of adolescents receiving cabotegravir LAI, with a median duration of approximately one day.^46^ Similarly, Simpson et al. reported discontinuation due to side effects in 11.5% (3/26) of pediatric patients receiving paliperidone LAI.^95^ These findings highlight the need to assess tolerability and acceptability alongside pharmacokinetics and efficacy during pediatric LAT development. Strategies to reduce procedure‐related burden should be incorporated early in product development and implementation planning. These may include age‐appropriate pain‐minimization approaches, careful selection of injection site and needle length, optimization of injection volume, child and caregiver education, and structured acceptability assessments. Minimally invasive delivery systems may be useful for selected pediatric LATs, while techniques such as Z‐track administration may reduce leakage and discomfort for some intramuscular injections, although suitability should be evaluated for each product, formulation, and age group.^96^

Regulatory considerations are also important. The development and approval of complex LATs, including LAIs, may require additional evidence on formulation characteristics, release kinetics, local tolerability, excipient safety, device compatibility, exposure matching, and post‐discontinuation pharmacokinetics. Existing US FDA pathways, including the 505(b)(2) New Drug Application and 505(j) Abbreviated New Drug Application routes, may be applicable in some circumstances for innovative and generic long‐acting products, respectively.^97^ However, pediatric LATs often require product‐specific evidence and early regulatory engagement, particularly when extrapolating from adult data (where plausible, e.g., adolescents dose selection) or adapting platform technologies across age groups. Greater regulatory clarity and harmonization would help innovators anticipate evidence requirements and support more efficient development of pediatric‐appropriate LATs.

## TARGET PRODUCT PROFILING FOR FUTURE PEDIATRIC LONG‐ACTING FORMULATIONS

Target product profiles (TPPs) are strategic tools used to align product development with clinical need, regulatory expectations, user requirements, and implementation context. WHO describes TPPs as documents that specify the intended use, target population, and preferred and minimally acceptable product characteristics, thereby guiding R&D priorities and supporting alignment among developers, funders, regulators, procurement agencies, and policy stakeholders.^98^, ^99^

For pediatric LATs, TPP development is particularly important because product success depends not only on efficacy but also on age‐appropriate formulation design, dose flexibility, route feasibility, excipient safety, tolerability, caregiver and child acceptability, storage requirements, cost, and implementation feasibility. **Table** **2** therefore proposes a cross‐cutting pediatric LAT attribute framework rather than a single disease‐specific TPP. The framework is intended to support future indication‐specific TPPs that can be tailored to disease context, therapeutic index, target age group, formulation platform, and health‐system setting.

**Table 2:** Proposed cross‐cutting product‐attribute framework for pediatric long‐acting therapeutic formulation development^a^

Attribute	Minimum acceptable characteristics	Preferred/optimal characteristics	Illustrative benchmark: cabotegravir/rilpivirine LAI^b^
Intended use/indication	Clearly defined pediatric indication with unmet need and plausible benefit from reduced dosing frequency or sustained exposure	Priority pediatric indication with strong public‐health relevance, clear clinical‐use case, and alignment with global or regional child‐health priorities	Treatment of HIV‐1 infection in virologically suppressed adolescents with no history of treatment failure and no known or suspected resistance to cabotegravir or rilpivirine
Target pediatric population	Specified pediatric age/weight group(s), including clear lower age and weight limits supported by available evidence	Age‐appropriate, non‐overlapping clinically meaningful pediatric groups, with a development plan spanning neonates, infants, children, and adolescents where relevant	Adolescents aged ≥12 years and weighing ≥35 kg
Efficacy benchmark	Efficacy target defined by indication and benchmarked against standard of care, accepted exposure‐response targets, or adult efficacy where extrapolation is justified	Efficacy comparable or superior to standard of care, with clinically meaningful advantage in adherence, prevention coverage, clinical outcomes, or implementation feasibility	High rates of virologic suppression reported in clinical studies; exposure and efficacy benchmarked against adult experience
Dose flexibility and dose banding	Weight‐ or age‐band dosing supported by pediatric PK data, modeling, or justified extrapolation	Simple age/weight bands covering intended pediatric groups with minimal dose manipulation and low monitoring burden	Fixed adult/adolescent dosing for those meeting age and weight criteria; younger pediatric dosing under evaluation
Duration of exposure/dosing interval	Clinically meaningful reduction in dosing burden vs. standard of care, with predictable exposure over the intended interval	Monthly or longer dosing interval where appropriate, with sustained therapeutic exposure and operational feasibility in routine care	Monthly or every‐2‐month IM maintenance regimens
Route and setting of administration	Age‐appropriate route feasible in intended care setting; administration requirements clearly defined	Route minimizes pain, visit burden, training needs, and health‐system complexity while maintaining reliable delivery	Intramuscular administration by trained healthcare personnel
Formulation and release characteristics	Formulation supports prolonged release with acceptable variability and manufacturable quality attributes	Predictable release profile, low variability, scalable manufacturing, and compatibility with pediatric dose volumes and administration devices	Nanosuspension LAI formulation administered as separate cabotegravir and rilpivirine injections
Initial burst/early exposure	Initial burst characterized and shown not to exceed safety thresholds in intended pediatric groups	Minimal clinically relevant burst release with early exposure within predefined safety margins	Initiation dosing and early post‐injection exposure are clinically characterised; excessive burst release is not a primary concern for this platform
PK tail and discontinuation strategy	PK tail characterized where relevant; missed‐dose and discontinuation management plan defined	Minimal clinically consequential PK tail or feasible cover/transition strategy, especially for anti‐infectives where resistance risk exists	Long terminal decline recognized; discontinuation requires transition to a fully suppressive alternative antiretroviral regimen
Systemic safety	Manageable adverse‐event profile with pediatric age‐group justification and monitoring plan	No serious or unexpected systemic safety concerns; low monitoring burden in routine use	No major unanticipated safety concerns reported in adolescent studies; safety monitoring remains required
Local tolerability and acceptability	Injection‐site or local reactions acceptable and manageable; pain and acceptability assessed in children/caregivers	Minimal local reactions and high child/caregiver acceptability; pain‐minimization strategy included from development stage	Mild‐to‐moderate injection‐site reactions occur and are generally manageable
Excipient burden and pediatric toxicology	Excipient exposure justified for intended age group, route, dose, and duration; toxicology gaps identified	Excipients with established pediatric safety experience at relevant doses/routes; minimal unnecessary excipient burden and age‐appropriate toxicokinetic data where needed	Excipients and formulation components characterized for the approved adolescent/adult product; younger pediatric extrapolation requires justification
Device or dosage‐form usability	Device/dosage form feasible for intended pediatric group and administrator; instructions support safe use	Child‐ and caregiver‐centered design; minimal training; low risk of administration error; compatible with outpatient or community delivery where appropriate	Healthcare worker administered IM injections; not designed for self‐administration
Drug–drug interactions and comorbidities	Relevant DDIs and comorbidity considerations identified for the pediatric indication and setting	Minimal clinically significant DDIs or clear dose‐adjustment/management guidance, including for common pediatric co‐treatments	Contraindications and interaction management required for antiretroviral co‐medications and other concomitant medicines
Monitoring requirements	Monitoring plan feasible in intended care setting and proportional to risk	Minimal specialized monitoring beyond routine clinical care, with clear management for missed doses and adverse events	Virologic monitoring and clinical follow‐up required; missed‐dose management specified
Shelf life and storage	Shelf life and storage compatible with intended use setting; cold chain needs justified where unavoidable	≥24–36 months shelf life where feasible; no cold chain or stability under relevant climatic‐zone conditions for LMIC deployment	Cold‐chain storage required for current product; shelf life product‐specific
Cost, access, and scalability	Cost and manufacturing approach compatible with intended procurement and health‐system setting	Affordable LMIC procurement pathway, scalable manufacturing, access strategy, and licensing/procurement plan considered early	Marked country‐level price variation; access depends on procurement, licensing, and implementation arrangements
Waste disposal and environmental considerations	Safe disposal plan for sharps, devices, packaging, or residual drug product	Minimal hazardous waste; biodegradable or low‐burden disposal strategy where feasible	Biohazard/sharps disposal required
Evidence generation and regulatory strategy	Pediatric evidence plan includes PK, safety, acceptability, and formulation/device considerations; early regulatory engagement considered	Model‐informed development plan, age‐appropriate trials, harmonized regulatory strategy, and post‐approval evidence generation for real‐world pediatric use	Approved through established regulatory pathways for defined adolescent/adult populations; pediatric expansion requires age‐specific evidence

DDI, drug–drug interaction; HIV, human immunodeficiency virus; IM, intramuscular; LAI, long‐acting injectable; LMIC, low‐ and middle‐income country; PK, pharmacokinetic; TPP, target product profile.

^a^ This table is intended as a flexible pediatric LAT attribute framework to support future indication‐specific target product profiles (TPPs), rather than as a single universal TPP for all diseases, age groups, routes, or platforms. Minimum and preferred characteristics should be tailored to the intended indication, therapeutic index, target age group, formulation platform, and implementation setting.

^b^ Cabotegravir/rilpivirine LAI is included as an illustrative benchmark for a clinically implemented long‐acting injectable platform. It should not be interpreted as the comparator for all pediatric LAT routes, technologies, diseases, or age groups.

Several attributes warrant particular emphasis. First, pediatric LATs should support clinically meaningful age or weight bands, because neonates, infants, children, and adolescents differ substantially in developmental physiology and dosing requirements. Second, safety and tolerability should include systemic safety, local tolerability, excipient burden, pain, device acceptability, and the feasibility of repeated or sustained administration.^73^, ^75^, ^100^ Third, LAT‐specific attributes such as release kinetics, initial burst, pharmacokinetic tail, reversibility, and discontinuation management should be incorporated, particularly for anti‐infective agents where prolonged subtherapeutic exposure may increase resistance risk. Finally, access‐related characteristics, including storage, shelf life, cost, training needs, supply‐chain feasibility, and feasibility in LMIC settings, should be considered from the outset rather than after product development is complete.^60^, ^69^, ^86^, ^87^

Existing pediatric TPP efforts, such as Drugs for Neglected Diseases Initiative's pediatric HIV TPP, illustrate the value of defining acceptable and ideal characteristics around target population, regimen, formulation, safety/tolerability, drug–drug interactions, stability, palatability, and cost. Similar principles should be adapted for pediatric LATs, while recognizing that no single platform or route will be optimal across all diseases and age groups. **Table** **2** should therefore be interpreted as a flexible framework for innovators, funders, policymakers, and regulators to prioritize pediatric‐appropriate long‐acting products and to develop more specific TPPs for individual indications.^98^, ^99^, ^101^, ^102^

## RECOMMENDATIONS BY THE PANEL

The continuing burden of pediatric disease, particularly infectious diseases that disproportionately affect children in LMICs, underscores the need for integrated prevention and treatment strategies.^1^, ^6^, ^7^ LATs could contribute to this agenda by sustaining therapeutic or prophylactic exposure, reducing dosing frequency, lessening caregiver burden, and supporting adherence where daily or frequent dosing is difficult.^35^ However, pediatric LAT development must be guided by child‐specific formulation needs, developmental pharmacology, implementation feasibility, and equitable access from the outset.^98^, ^99^

First, pediatric LAT development should be aligned with clearly defined disease priorities and use cases. Early target product profile development should specify the intended pediatric age groups, route of administration, dose flexibility, storage and supply‐chain requirements, affordability, and suitability for LMIC deployment.^98^, ^99^ Product development should not be driven only by technical feasibility or high‐income‐market opportunity, but by the potential to address priority pediatric conditions where adherence, access, or health‐system constraints limit current care.

Second, developmental pharmacology and pediatric formulation science should be embedded from the earliest stages. Children should not be treated as small adults: neonates, infants, children, and adolescents differ in body composition, tissue characteristics, organ maturation, absorption, clearance, and tolerability.^37^, ^39^, ^56^, ^57^ Dose selection should therefore be supported by pediatric pharmacokinetic data, population PK and PBPK modeling, and age‐ or weight‐band dosing strategies where appropriate.^38^, ^60^

Third, the panel recommends development of a standardized protocol for assessing acceptability of pediatric LAT formulations and technologies. Such a protocol should be age‐appropriate and should capture child, caregiver, and healthcare‐provider perspectives on route preference, pain, anxiety, local reactions, device burden, dosing interval, clinic‐visit burden, caregiver administration, and willingness to continue therapy. For non‐injectable LATs, the protocol should also assess issues such as patch adhesion, device retention, removability, swallowing ability, and day‐to‐day usability. Pediatric acceptability assessment is increasingly recognized as important for adherence and successful medicine development, and emerging methods support the use of child‐centered and composite acceptability approaches.^103^

Fourth, LAT‐specific risks should be explicitly evaluated in pediatric development plans. These include initial burst release, prolonged pharmacokinetic tails, discontinuation strategies, missed‐dose management, excipient burden, local tissue tolerability, and resistance risk for long‐acting anti‐infective agents.^41^, ^45^, ^65^, ^66^, ^73^, ^74^ For anti‐infectives, developers should define how subtherapeutic tail concentrations will be monitored and managed, including whether oral cover or transition therapy is required after discontinuation.^41^, ^45^

Fifth, regulatory pathways for pediatric LATs should be clarified and harmonized where possible. Complex long‐acting formulations may require evidence beyond conventional immediate‐release products, including formulation characterization, release kinetics, pediatric exposure matching, device compatibility, local tolerability, excipient safety, and post‐discontinuation pharmacokinetics.^98^ Early engagement among developers, regulators, pediatric experts, and global‐health stakeholders would help define proportionate evidence requirements and reduce uncertainty.

Finally, equitable access should be treated as a core design requirement rather than a post‐approval consideration. Without deliberate planning, LATs could widen health inequalities if they are developed, priced, or distributed primarily for well‐resourced settings while the highest pediatric disease burden remains in LMICs.^98^, ^99^ Developers, funders, procurement agencies, governments, regulators, global‐health organizations, and community stakeholders should work together to support affordable pricing, scalable manufacturing, appropriate licensing, sustainable supply chains, feasible storage conditions, and implementation models that reach children with the greatest need. WHO GAP‐f may provide an important platform for aligning pediatric health priorities, formulation development, regulatory expectations, and access planning.

## CONCLUSIONS

Successful development and implementation of pediatric long‐acting therapeutics will require a coordinated, child‐centered, and equity‐driven approach that integrates formulation science, developmental pharmacology, model‐informed dosing, standardized acceptability assessment, regulatory planning, and early access considerations.^2^, ^3^ LATs have the potential to reduce treatment burden, improve adherence, sustain therapeutic or prophylactic exposure, and address priority pediatric health needs, particularly where frequent dosing or health‐system barriers limit current care. Realizing this potential will depend on products that are age‐appropriate, acceptable to children and caregivers, safe across developmental stages, affordable, scalable, and feasible in the settings where need is greatest.

## FUNDING

This work was supported by Unitaid (grant number 2020–38‐ LONGEVITY). AO receives funding from the Wellcome Trust (227288/Z/23/Z) and United States NIH (through LEAP award number 2R24AI118397–11). DH receives funding from National Institute of Health and Care Research (through NIHR Alder Hey Clinical Research Facility). The views expressed are those of the authors and not necessarily those of the funders. For open access, the authors have applied a CC‐ BY public copyright license to any Author Accepted Manuscript version arising from this submission.

## CONFLICT OF INTEREST

All authors declared no competing interests for this work.

## USE OF ARTIFICIAL INTELLIGENCE (AI)

All authors confirm that no AI tools were utilized for drafting or for the creation of figures and tables in this article.
